# Support for Adjusting the Intentions of Family Members and Users Regarding Care Service Use: Aimed at Care Management

**DOI:** 10.1093/geroni/igab046.3092

**Published:** 2021-12-17

**Authors:** Kazutaka Masuda

**Affiliations:** Mukogawa Women’s University, Nishinomiya, Hyogo, Japan

## Abstract

In Japan, there are key healthcare professionals for home nursing care for elderly people called Care Managers. The care manager coordinates the service while adjusting the family situation and the user's intentions. The purpose of this study was to examine the practical structure of support for adjusting the intentions of family members and users regarding care service use. Data from seven cases, where family members and users have different intentions regarding care service use, were analyzed using the grounded theory approach. The phenomenon of "confirmation of discrepancies" was discovered with six sub-categories: adjusting the intentions of users and their families, effort to restore relationships, expression of intention to refuse involvement, expression of desire for adjustment, arrangement of opportunities for adjustment of intentions, and appropriate service adjustment. Four patterns occurred in the process of "confirmation of discrepancies": smooth adjustment, restoration and promotion of mutual relationships, failure to reach an agreement, and negative feedback loops. These patterns were based on a combination of the care managers' degree of understanding strength, the managers' degree of insistence, the managers' degree of representation of mutual feelings, the degree of managers' prediction of life prospects, the degree of trust in care managers, and the degree of expression of family anxiety.

